# Myositis Ossificans of the Soleus Muscle

**DOI:** 10.7759/cureus.100788

**Published:** 2026-01-04

**Authors:** Sofia Kada, Saadia Ait Malek, Erraoui Mariam, Imad Ghozlani

**Affiliations:** 1 Rheumatology, University Hospital of Mohammed VI, Agadir, MAR; 2 Rheumatology, Faculty of Medicine and Pharmacy of Agadir, Ibn Zohr University, Agadir, MAR; 3 Rheumatology, Cartilage and Bone (CARBONE) Research Team, Research Laboratory of Innovation in Health sciences (LARISS), Faculty of Medicine and Pharmacy of Agadir, Ibn Zohr University, Agadir, MAR; 4 Rheumatology, Oued Eddahab Military Hospital, Agadir, MAR

**Keywords:** a rare entity, clinical case report, myositis ossification, physical therapy rehabilitation, post trauma, soft-tissue calcification, soleus muscle, surgical removal

## Abstract

Myositis ossificans (MO) is a rare condition in which abnormal calcification and bone formation develop within a muscle. It appears most often in adolescents but can also occur in younger children. Although it usually affects a single muscle, it can sometimes involve different parts of the body.

We report a case of post-traumatic MO. A 16-year-old girl presented with progressive left knee pain and swelling 15 days after minor trauma, with a visual analog scale (VAS) for pain of 8/10 and nocturnal exacerbation. Examination showed a soft, mobile, tender medial knee mass with reduced range of motion. Laboratory tests revealed elevated erythrocyte sedimentation rate and serum alkaline phosphatase with normal C-reactive protein and creatine phosphokinase.

Ultrasound demonstrated heterogeneous thickening of the semimembranosus tendon with a 20×17 mm hypoechoic peritendinous area, consistent with peripheral myo-aponeurotic avulsion. Positron emission tomography imaging showed a hypermetabolic calcified extraosseous lesion adjacent to the medial tibial plateau without cortical disruption, raising concern for MO, organized hematoma, or malignancy (notably osteosarcoma). Computed tomography confirmed mixed calcifications (26×24 mm) with heterogeneous enhancement. Magnetic resonance imaging revealed an ovoid calcified soft-tissue mass within the soleus/paratibial region, marked surrounding edema, and mild periosteal reaction, supporting an intermediate-stage juxtacortical MO and making a primary bone tumor unlikely.

Management included nonsteroidal anti-inflammatory drugs, ketoprofen, then indomethacin, zoledronic acid infusion, and physical therapy emphasizing pain-free mobilization and ultrasound therapy.

## Introduction

Myositis ossificans (MO) is a rare musculoskeletal condition characterized by heterotopic calcification and ossification of muscular tissue that predominantly occurs in adolescents, although cases have also been documented in early childhood. The disease is often restricted to a single muscle but may involve various parts of the body.

The condition can arise from several factors, such as genetic predisposition and post-infectious inflammation, with tissue injury playing a key role in its development [1,2].

This clinical entity is well defined, and it can be classified into three major forms: progressive ossifying myositis, also known as fibrodysplasia ossificans progressiva (FOP), post-traumatic or circumscribed myositis ossificans (TMO), and atraumatic or pseudo-malignant form [3]. TMO is the predominant form of the disease, which represents almost 60-75% of the cases [1]. 

Various muscles were reported in published cases, including iliopsoas, triceps brachii, quadriceps femoris, biceps femoris, tibialis anterior, paravertebral, buccinators, masseter, etc. [4].

We present the case of a 16-year-old girl with consistent and progressive pain in the left knee after a domestic accident and her imaging findings revealed ossification of the left soleus muscle.

## Case presentation

A 16-year-old girl was admitted to our hospital complaining of a painful knee after a domestic accident three months earlier. The patient fell from standing height after slipping on a wet floor, and 15 days after the trauma, she developed a painful swelling on the medial compartment of the left knee, extending along the left lower limb with progressive worsening of symptoms and a visual analog scale (VAS) for pain of 8/10. The patient also experienced a nocturnal exacerbation of pain leading to functional decline.

Physical examination reveals an apyretic patient with a well-circumscribed, soft, mobile, tender swelling with a decreased range of motion in the left knee: A limited extension to 20° and a maximal flexion of 80°.

Laboratory tests showed an elevated erythrocyte sedimentation rate (ESR) and serum alkaline phosphatase (SAP) with a normal C-reactive protein (CRP) and creatine phosphokinase (CPK) (Table 1).

**Table 1:** Laboratory test findings with reference ranges ESR: Erythrocyte Sedimentation Rate; CRP: C-reactive Protein; SAP: Serum Alkaline Phosphatase; CPK: Creatine Phosphokinase

Laboratory tests	Results	Reference ranges
ESR	53mm/1^st^ hour	<20mm/1^st^ hour
CRP	0.5mg/L	<6mg/L
SAP	200UI/L	30-130UI/L
CPK	105UI/L	60-140UI/L

The ultrasound examination of the left knee revealed a heterogeneous thickening of the semimembranosus tendon surrounded by a focal hypoechoic area measuring 20x17mm, suggesting a peripheral myo-aponeurotic avulsion.

**Figure 1:**
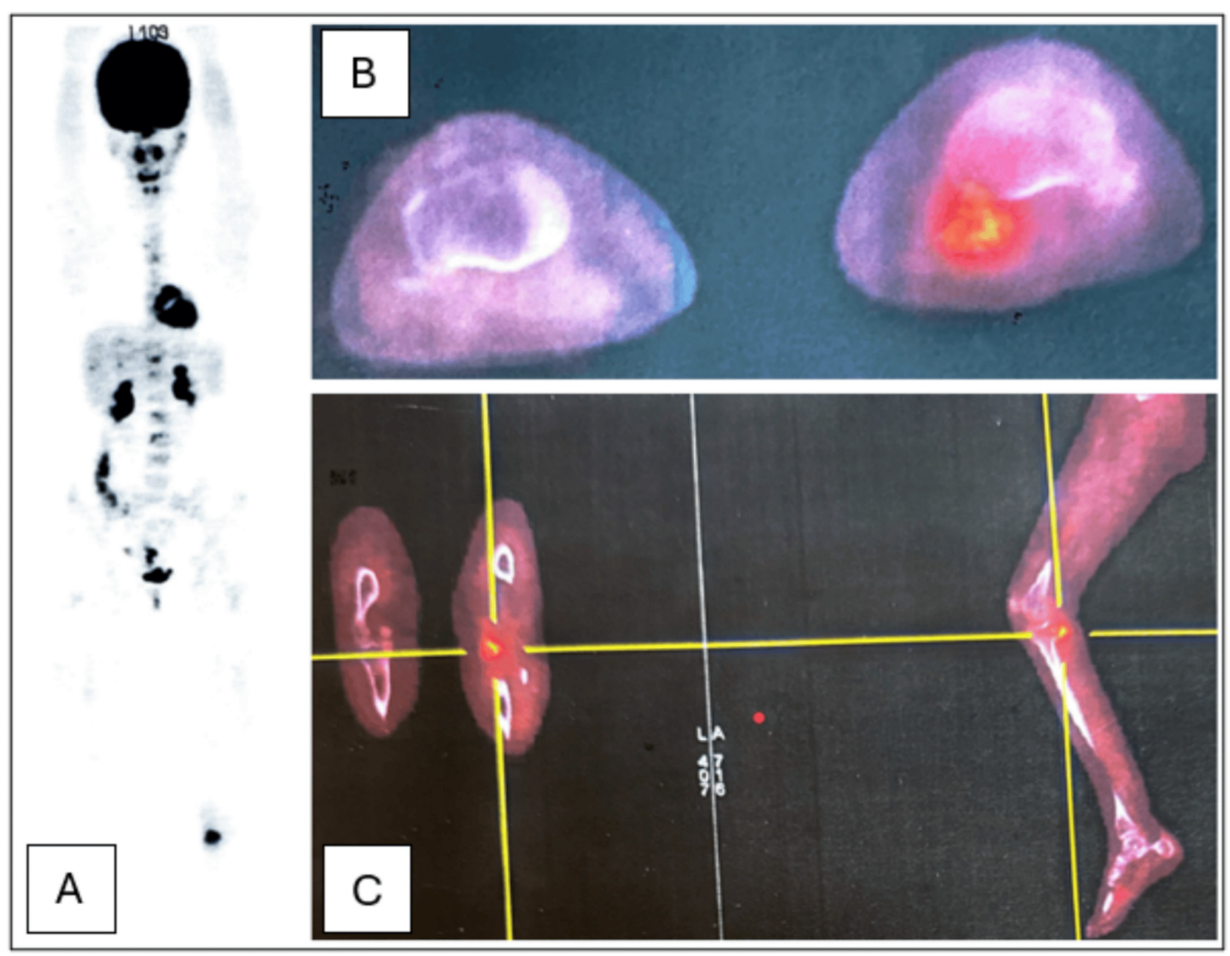
Imaging findings on a PET scan A: PET imaging demonstrating a hypermetabolic calcified lesion of the left leg; B: Axial PET imaging of the calcified lesion with close proximity to the tibial cortex without any cortical breach or osseous involvement; C: PET imaging showing a hypermetabolic calcified lesion on the posterior surface of the medial tibial plateau

PET imaging demonstrated a hypermetabolic calcified lesion on the posterior surface of the medial tibial plateau, with close proximity to the cortex without any cortical breach or osseous involvement. These findings suggested an extraosseous pathology potentially related to MO or a subacute organized hematoma. At this stage, a malignant etiology was suspected as well, particularly osteosarcoma which led us to further investigations (Figure 1).

The CT revealed mixed central and peripheral distribution of calcifications, the largest measuring 26x24mm, and heterogeneous contrast enhancement with a significant central accentuation.

**Figure 2:**
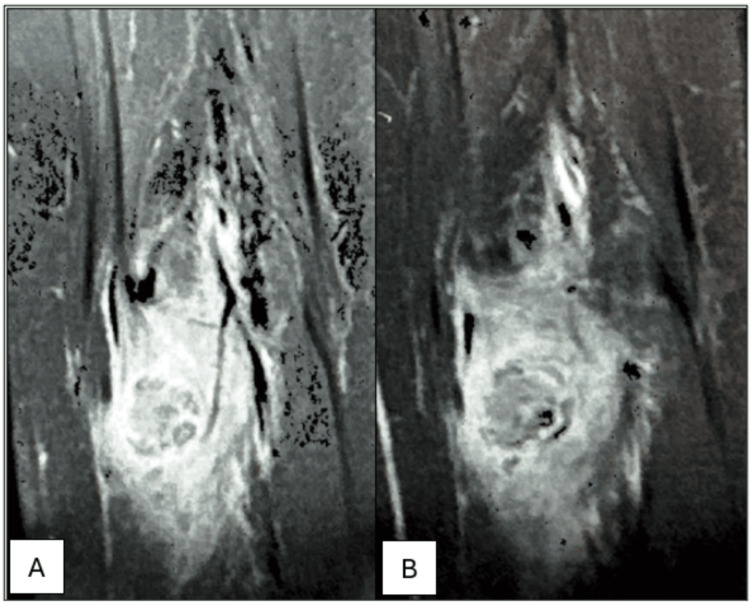
Imaging findings on MRI. A: T1-weighted sequence; B: T2-weighted sequence MRI findings showing a calcified ovoid mass within the soleus muscle, with indistinct margins and heterogeneous signal on both T1 and T2-weighted sequences. A significant inflammatory muscle and bone marrow edema, as well as cortical irregularities with periosteal reaction suggesting an intermediate-stage juxtacortical MO MO: Myositis ossificans

MRI findings showed an intermediate-stage juxtacortical MO of the soleus muscle (Figure 2). A primary bone tumor remained unlikely.

Pharmacological management involved an initial course of ketoprofen (100mg), which we switched to indomethacin (100mg) following the lack of clinical improvement, in addition to zoledronic acid infusion and physical therapy based on infra-painful mobilization to maintain joint range of motion and muscle strength, combined with ultrasound therapy.

## Discussion

MO is a rare pathological entity defined by heterotopic ossification (HO) within soft tissues, with a predilection for skeletal muscle. Approximately 75% of MO cases are attributed to trauma, while the remaining 25% are attributed to neurogenic, congenital, or idiopathic etiologies [4]. It can occur at any age but it is rarely observed in both early and advanced age and occurs more frequently in men. The youngest documented case involved a 17-day-old infant while the oldest concerned an 81-year-old woman [3].

Epidemiology

MO can be classified into three distinct types based on hereditary factors: Traumatic or circumscribed MO, progressive MO or FOP, and non-traumatic or pseudo-malignant MO [4]. The latter includes cases associated with conditions such as burns, polio, paraplegia, hemophilia, or infections. TMO is the most frequently encountered form and is typically associated with a documented history of musculoskeletal trauma [4]. MO most frequently involves the diaphysis of long bones and within the major muscle groups, particularly the brachialis, quadriceps, and adductor muscles [5]. Its presence in the paravertebral and cervical region is rare, typically presenting as a unilateral manifestation in most cases [3].

Physiopathology

The physiopathological mechanisms underlying MO remain poorly elucidated, although several hypotheses have been advanced. It is generally believed that the lesion results from aberrant differentiation of fibroblasts into osteogenic lineages. This process is typically initiated by acute skeletal muscle trauma, triggering a localized inflammatory cascade characterized by the release of various cytokines, particularly bone morphogenetic proteins (BMP-2 and BMP-4) and transforming growth factor-beta (TGF-β) [5].

These signaling molecules stimulate perivascular mesenchymal cells, promoting their differentiation into osteoblasts and chondroblasts. Some of these differentiated cells subsequently contribute to endochondral bone formation within extraskeletal soft tissue.

Additionally, muscle injury leads to increased synthesis of proinflammatory prostaglandins, which further facilitate HO. In trauma-associated cases involving hematoma formation, secondary processes including tissue necrosis, hemorrhagic infiltration, and subsequent fibrovascular proliferation are thought to exacerbate ectopic ossification.

TMO follows a progression through three distinct stages: early, intermediate and mature. Each stage is defined by specific radiographical and histological features. Although exact timelines may vary across individuals and studies, the early phase typically occurs within the first four weeks following trauma, the intermediate phase from approximately week four to week eight, and the mature phase beyond eight weeks, potentially extending over several months [5].

Histology

In the early stage of MO, histology reveals a proliferation of fibroblasts and myofibroblasts, with minimal osteoid formation. This phase is marked by increased mitotic activity that results in spindle cells and hyperchromatic nuclei and may mimic high-grade sarcomas. Therefore, this stage is commonly designated as the “pseudosarcomatous stage”, highlighting the risk of misdiagnosis in the absence of clinical and radiological correlation [5].

A typical MO lesion is characterized by three distinct histological zones. The central zone is populated by proliferating fibroblasts with variable areas of hemorrhage and necrosis. The intermediate zone is predominantly composed of osteoblasts involved in the formation of immature osteoid tissue. The peripheral zone is primarily made up of mature, well-organized lamellar bone, reflecting progressive maturation and ossification from the center outward. A key differentiating feature lies in the pattern of mineralization: osteosarcomas typically demonstrate a centripetal ossification pattern, whereas MO exhibits a centrifugal maturation of calcified tissue [5].

Clinical presentation

The clinical presentation, as with the histological features, is stage dependent in the evolution of MO (Table 2). The typical presentation is that of a rapidly enlarging, painful, and inflammatory soft tissue mass, often mimicking a soft tissue sarcoma in its early stages. Within a few weeks, the lesion becomes firm and may remain tender. Over a period of 6 to 12 months, the mass undergoes ossification, typically becoming a well-circumscribed, asymptomatic mass [6].

**Table 2:** Characteristics of myositis ossificans during each stage Created by authors based on the text from [5]

Stage	Presentation	Histopathology
Early (0-4 weeks)	Possible traumatic history; localized pain and swelling; decreased range of motion	High fibroblast/myofibroblast count with high mitotic activity; very minor osteoid formation; centrifugal zonal pattern develops.
Intermediate (4-8 weeks)	Persistent pain and decreased range of motion; possible soft tissue mass palpable	The centrifugal zonal pattern disappears; osteoid core and peripheral mature bone
Mature (>8 weeks)	Improved or resolved pain and range of motion; possible soft tissue mass palpable	Diffuse lamellar bone

The development of MO should be suspected when pain persists beyond the expected resolution period of an otherwise uncomplicated muscle strain or contusion. Typically localized to the site of previous trauma, the pain may be accompanied by restricted joint mobility or stiffness in adjacent joints. Involvement of the proximal thigh may additionally present functional limitations such as impaired weight-bearing and reduced knee flexion. Clinical findings may include localized tenderness, ecchymosis, and soft tissue swelling, particularly in the anterior thigh.

Given that MO lesions can arise in any skeletal muscle, there is potential for involvement and compression of adjacent neurovascular structures, leading to more concerning manifestations such as muscle weakness, paresthesia, lymphedema, or even venous thromboembolic events. Typically, the lesion undergoes a phase of active proliferation for approximately 10 weeks, after which it enters a quiescent, painless stage, followed by gradual spontaneous regression [7].

Laboratory testing

Laboratory findings similarly fluctuate according to the maturation of MO. In the early inflammatory phase of MO, elevations in nonspecific acute-phase reactants such as CRP, ESR and prostaglandin E2 may be elevated. SAP, a marker of osteoblastic activity, typically remains within normal limits during the first three weeks following acute muscle injury. However, as HO progresses, SAP levels may rise in parallel with lesion maturation, generally peaking around ten weeks and returning to baseline by approximately 18 weeks [8]. Despite this temporal association, SAP levels do not reliably correlate with the degree of lesion maturity or metabolic activity, limiting their utility in staging MO. Moreover, the diagnostic specificity of SAP is restricted, as elevated levels can also be encountered in other pathologies, such as osteosarcoma.

In addition, serum calcium levels may experience a mild transient decrease shortly after the initial injury but typically normalize within three weeks, preceding the rise in SAP levels.

There is currently no scientific evidence demonstrating a direct correlation between CPK levels and the risk of developing MO. However, certain inferences may be drawn from the observations of Singh et al., who demonstrated that CPK levels are consistently elevated following skeletal muscle injury and, in contrast to SAP, may constitute a potential biomarker for predicting both the onset and the clinical severity of MO [5].

Imaging

A variety of imaging modalities may be used to establish the diagnosis of MO and to differentiate it from malignant soft-tissue neoplasms. In the acute post-traumatic phase, CT represents the most sensitive technique for detecting early soft-tissue changes, prior to the appearance of mineralization on conventional radiography.

During this initial period, calcific deposits are typically absent, as the lesion is predominantly composed of proliferating myofibroblasts and immature connective tissue resembling granulation tissue. Between approximately two and six weeks following the inciting trauma, plain radiographs may demonstrate a central radiolucent zone surrounded by a well-defined peripheral rim of ossification. At more advanced stages, typically after six months, the lesion acquires the characteristic appearance of a circumferential calcified shell oriented parallel to the long axis of the adjacent bone. This mature ossified margin is separated from the underlying cortical bone by a distinct radiolucent interface, referred to as the “string sign” [9].

CT is considered the gold standard imaging technique for the evaluation of MO. In the immediate post-traumatic phase, CT typically reveals soft tissue swelling without evidence of calcification. Over the ensuing four to six weeks, progressive peripheral mineralization becomes evident, manifesting as the characteristic zonal or “string sign” pattern indicative of early ossification. Moreover, CT is the preferred modality for follow-up imaging and plays a crucial role in differentiating MO from osteosarcoma by delineating the specific zonal maturation pattern [10].

MRI provides limited diagnostic specificity in the assessment of MO, as its primary role is confined to the early post-traumatic phase. Within approximately the first eight weeks following injury, MRI may demonstrate nonspecific findings such as bone marrow edema, periosteal reaction, intermuscular fluid collections, and reactive joint effusion, all of which reflect acute inflammatory and reparative processes. However, MRI is inherently limited in its ability to depict the progressive mineralization and peripheral zonal ossification that are pathognomonic of MO. In the chronic or mature stage, ultrasonography constitutes a more suitable modality, allowing visualization of a well-defined lesion characterized by a continuous, highly echogenic, calcified peripheral rim [11].

Bone scintigraphy represents an additional imaging modality that may be employed for the evaluation of MO. Although it is highly sensitive, it lacks specificity for distinguishing this entity from other calcified soft-tissue lesions. In the early stages of the disease, scintigraphy typically demonstrates increased radiotracer uptake corresponding to active osteoblastic activity, which gradually normalizes as the lesion matures and ossification stabilizes. This technique is primarily indicated when surgical excision of the lesion is necessary, as it assists in determining the optimal timing for intervention. Serial bone scans demonstrating a progressive reduction in radiotracer accumulation are indicative of lesion quiescence and maturation, thereby suggesting a lower risk of recurrence following resection [9].

Overall, each imaging technique has its own particularities in the detection of MO according to the stage of maturation, as summarized in Table 3.

**Table 3:** Imaging findings in myositis ossificans Created by authors based on the text from [9]

Phase	Early	Intermediate	Mature
Plain film	< two or four weeks; soft tissue swelling; faint peripheral calcification	Four weeks to six months; well-defined peripheral calcification; coarser central calcification may be present	> six months; densely calcified lesion; usually parallel to the long axis of the adjacent bone
Ultrasound	Hypoechoic soft tissue mass with hyperechoic core; fluid levels (hemorrhage) may be present (nonspecific finding)	Peripheral lamellar calcification	Highly reflective, heavily calcified; rim may be irregular due to lesion shrinkage
Computed Tomography scan	Increased tracer uptake on all phases; soft tissue swelling; faint calcification may be present	Decreasing tracer uptake; peripheral calcified rim; central zone isodense to muscle	Normal/ mildly increased uptake; dense calcification of the lesion
Magnetic Resonance Imaging	Intramuscular nodule/swelling; isointense T1; hyperintense T2; peripheral or general enhancement; may be faint low signal foci or rim	Variable central signal; low signal intensity rim and central foci in all sequences; variable pattern of enhancement; reduced perilesional; T2-hyperintensity (edema)	Generally low signal on all sequences; no perilesional edema

Management

Given that most cases of MO are self-limiting and undergo spontaneous resolution, conservative management is generally considered the most appropriate initial management approach. The fundamental principle guiding MO treatment is the alleviation of symptoms most notably pain, and the restoration of normal function and range of motion.

Early management of traumatic muscle injury adheres to the established RICE protocol (Rest, Ice, Compression and Elevation), which aims to prevent or limit intramuscular hemorrhage and subsequent hematoma formation, which represents a key predisposing factor in the subsequent development of HO [5].

The use of nonsteroidal anti-inflammatory drugs (NSAIDs) for analgesia remains a subject of debate, particularly during the acute phase, as these agents may potentiate the risk of recurrent or exacerbated bleeding. Indomethacin is the most extensively studied, although naproxen and diclofenac appear to demonstrate comparable efficacy in preventing HO.

The prophylactic effect of NSAIDs on HO is attributed to the inhibition of prostaglandin synthesis and, potentially, to the modulation of pre-osteoblast differentiation. When indomethacin is used, a dosage of 25-50 mg administered two to three times daily for seven to 11 days is generally recommended [12]. Considering the available evidence, NSAIDs may therefore be advocated as a preventive strategy for MO. Naproxen, ibuprofen, and indomethacin are all viable options; however, indomethacin remains the preferred agent, as it is the most thoroughly investigated NSAID in the context of HO prevention [10].

Cryotherapy, applied for 15-20 minutes at intervals of 30-60 minutes, may reduce regional blood flow to the affected area by up to 50%. A brief period of immobilization, typically lasting three to seven days and supported by crutch use, may further mitigate hematoma development. Initiating aggressive rehabilitation protocols prematurely can aggravate symptoms and should be avoided. Conversely, gentle, assisted range-of-motion exercises may be initiated as early as 48-72 hours post-injury. Mild discomfort is acceptable during early mobilization; however, significant pain should prompt reassessment of the rehabilitation approach [6].

Additional nonoperative management strategies include extracorporeal shockwave therapy (ESWT), ultrasound-guided hematoma aspiration, and pharmacologic prophylaxis preventing MO following muscle injury. A 2017 case report described a 15-year-old male soccer player presenting with an MO lesion of the vastus intermedius who underwent a protocol of five weekly ESWT sessions, initiated at eight weeks post-injury. The patient reported a notable reduction in pain and an improvement in joint range of motion after only two treatment sessions. By week 12, full knee range of motion had been restored, permitting the initiation of light jogging, and the athlete achieved unrestricted return to competitive play by week sixteen [13]. Current evidence does not support hematoma aspiration as an effective preventive measure for MO; given its invasiveness, it should be reserved for selected cases.

Pharmacologic prophylaxis also lacks strong evidence, with most data extrapolated from studies in pelvic trauma and hip surgery. A single case report described clinical and radiographic improvement after two doses of pamidronate in an athlete with traumatic MO. Etidronate has shown benefit in treating HO after spinal cord injury, but there is little evidence supporting bisphosphonate use for MO following muscle contusion. Bisphosphonates act by binding to hydroxyapatite, reducing bone turnover and slowing mineralization. In one reported case, pamidronate treatment lowered elevated bone turnover markers such as cross-linked N-telopeptide (NTx) and SAP to normal levels for age. These findings suggest that intravenous bisphosphonates may offer a potential off-label option for managing MO, though further research is needed to confirm their safety, optimal dosing, and effectiveness, particularly in children. Despite growing interest in injection-based therapies, no evidence supports the use of corticosteroids, proliferative agents, or platelet-rich plasma for MO management [14].

Mature MO lesions are usually painless, with inflammation resolving and possible spontaneous regression. Surgical excision is reserved for persistent, symptomatic cases after failed conservative therapy. Most authors recommend delaying surgery for at least six months to reduce recurrence by ensuring lesion maturation. Orava et al. reported good outcomes, with most athletes returning to preinjury level. Early excision may also be safe, though evidence remains limited [15].

## Conclusions

In conclusion, we reported a rare case of MO which is a benign, self-limiting ossifying lesion within skeletal muscle, often following blunt trauma. Early lesions can mimic malignancy, particularly osteosarcoma, but the characteristic zonal maturation pattern aids differentiation. Radiographs are the first-line imaging, with ultrasound increasingly used for diagnosis and follow-up. MRI provides superior soft tissue detail but has limitations. Management is primarily conservative; surgical excision is reserved for persistent pain or functional limitation, typically delayed until lesion maturation. Further research is required to better understand its pathophysiology and thereby elucidate more effective therapeutic approaches for this rare condition.
